# A Socio-Ecological Approach to Addressing Digital Redlining in the United States: A Call to Action for Health Equity

**DOI:** 10.3389/fdgth.2022.897250

**Published:** 2022-07-18

**Authors:** Terika McCall, Kammarauche Asuzu, Carol R. Oladele, Tiffany I. Leung, Karen H. Wang

**Affiliations:** ^1^Division of Health Informatics, Department of Biostatistics, Yale School of Public Health, New Haven, CT, United States; ^2^Center for Medical Informatics, Yale School of Medicine, New Haven, CT, United States; ^3^Yale Child Study Center, Yale School of Medicine, New Haven, CT, United States; ^4^Equity Research and Innovation Center, Department of Internal Medicine, Yale School of Medicine, New Haven, CT, United States; ^5^Care and Public Health Research Institute/Maastricht University Medical Center, Maastricht, Netherlands; ^6^Department of Internal Medicine (Adjunct), Southern Illinois University School of Medicine, Springfield, IL, United States

**Keywords:** health equity, digital health equity, social determinants of health, internet access, digital redlining, COVID-19

## Abstract

Physical distancing requirements due to the coronavirus (COVID-19) pandemic has increased the need for broadband internet access. The World Health Organization defines social determinants of health as non-medical factors that impact health outcomes by affecting the conditions in which people are born, grow, work, live, and age. By this definition broadband internet access is a social determinant of health. Digital redlining—the systematic process by which specific groups are deprived of equal access to digital tools such as the internet—creates inequities in access to educational and employment opportunities, as well as healthcare and health information. Although it is known that internet service providers systematically exclude low-income communities from broadband service, little has been done to stop this discriminatory practice. In this paper, we seek to amplify the call to action against the practice of digital redlining in the United States, describe how it contributes to health disparities broadly and within the context of the COVID-19 pandemic, and use a socio-ecological framework to propose short- and long-term actions to address this inequity.

## Introduction

“Of all the forms of inequality, injustice in health is the most shocking and the most inhuman because it often results in physical death.”—Dr. Martin Luther King Jr.

More than 50 years since Dr. King's speech, significant health disparities persist in lower income communities of color in the United Sates. Social determinants of health (SDH) primarily drive these health disparities. SDH are non-medical factors that impact health outcomes by affecting the “conditions in which people are born, grow, work, live, and age” (1). Examples include access to safe and affordable housing, educational and employment opportunities, and healthy foods, which account for 80% of health outcomes (2). Frequently overlooked as a SDH, access to broadband internet also emerged as essential infrastructure particularly during the COVID-19 pandemic (3–5). For example, various regulations enacted to mitigate community transmission of COVID-19 meant that broadband internet access became essential for people working remotely, online or distance learning, and virtual health visits *via* video or phone. Some communities, such as Black and Brown communities (6), have been disproportionately affected by the pandemic, exacerbating the existing digital divide that includes but is not limited to broadband internet access.

In this paper, we aim to increase awareness about the practice of digital redlining in the United States, its health consequences, and amplify the call to action for public health experts and advocates to support essential communications and technology service infrastructure, such as access to broadband internet, internet-enabled digital devices, and efforts to increase digital literacy in under-resourced communities. Specifically, we (a) discuss digital redlining and the impact lack of broadband internet access had on education access and quality, employment, and access to high-quality health care in the context of the COVID-19 pandemic; (b) apply a socio-ecological framework to propose synergistic short- and long-term strategies to expand broadband internet access; and (c) present existing work focused on addressing this inequity. Our perspectives draw from personal and professional experiences. We have developed digital health solutions for people who are underserved by mental health resources (7). Our patients, research study participants, family, and friends have experienced inability to participate in distance learning, inability to work remotely, or limited ability to access virtual care and telecounseling—primarily due to insufficient broadband internet access.

## Digital Redlining and the Impact of the COVID-19 Pandemic

*Redlining* originally described a discriminatory practice in which lenders classified neighborhoods, predominantly Black and Brown communities, as risky investments and therefore denied loans to individuals wanting to purchase homes in these neighborhoods (8, 9). The Fair Housing Act of 1968 made the practice of redlining unlawful (10). However, the effects of redlining can still be felt today in many lower income communities of color: communities that were redlined endured disinvestment, resulting in food deserts, limited access to educational and employment opportunities, and fewer options to receive quality health care (11, 12).

*Digital redlining*, or the intentional lack of investment in broadband infrastructure and affordable service offerings in low-income communities, contributes to health inequities (13–15). A study exploring historical redlining and broadband internet access in Milwaukee showed inequities in broadband access prior to the COVID-19 pandemic; neighborhoods rated the highest “A” (predominantly White and higher income households) were significantly more likely to have broadband access than “D” rated neighborhoods (historically redlined lower income communities of color), 82 vs. 62% (15). Similarly, a study conducted by the Greenlining Institute discovered that areas in California that were redlined in the past (e.g., Oakland) currently lack sufficient broadband internet access (16).

The World Health Organization declared a global COVID-19 pandemic in March 2020 (17), as a result broadband internet access became a necessity. Low-income earners, individuals living in rural communities, elderly, and Black and Brown individuals were less likely to have broadband internet services and more likely to access the internet using their mobile devices (18). Approximately 45% of lower income households (< $25,000) and around 36% of rural households do not have access to broadband internet (19). Furthermore, the lack of access to broadband internet disproportionately affected Black (31%), Latino (31%), and American Indian/Alaska Native (34%) children (19). These communities relied on mobile device data for internet-based services during the pandemic, including schooling, work, and virtual health visits (18). Inequities related to access to quality education, employment, and health care seen pre-pandemic were worsened by no or limited access to broadband internet.

### Education

Education access and quality is a SDH: higher levels of education have been linked to better health and longer life-span (20). Higher education is a pathway to obtaining higher paying jobs to improve standard of living and accumulate generational wealth. During the COVID-19 pandemic, the abrupt shift to remote learning has disadvantaged ~16.9 million children in the U.S. who did not have access to broadband internet (19). Children unable to engage fully in distance learning experienced a “homework gap”—difficulty participating in remote learning and completing assignments—due to lack of broadband internet and inadequate computer or device access at home (21).

Children also experienced a widening achievement gap during the pandemic. For example, students in grades 8–11 without broadband internet access took longer to complete assignments and had lower grade point averages (22). Some students even sat in parking lots outside of schools, libraries, and restaurants to access internet for remote learning (23, 24). The disparity in broadband internet access is seen across education levels. College students in rural areas, lower income and Latino households were less likely to have broadband internet access at home than their counterparts (25). Individual workers with higher education levels were more likely to be able to transition to working remotely during the pandemic, reducing their possible COVID-19 exposure.

### Employment

Limited educational opportunities translate into limited employment opportunities downstream. Access to safer and higher paying jobs are well-recognized SDH that were exacerbated by broadband internet access disparities during the COVID-19 pandemic. COVID-19 regulations classifying essential workers also systematized inequities by occupation. That is, possible exposure to COVID-19 varied by occupation, leading to exposure risk disparities because individuals from minoritized communities (e.g., Black and Latino) are more likely to occupy *high exposure risk* jobs (e.g., industrial, retail, and transit jobs) (26)—occupations involving close contact, within six feet, of another individual for a total of 15 min or more over a 24-h period (27).

In contrast, *lower exposure risk* occupations do not require close contact (27). This includes remote workers, office workers who do not have frequent close contact with coworkers, customers, or the public, and healthcare workers providing telehealth services only. Individuals who could pivot to remote work by utilizing broadband internet not only reduced their risk of COVID-19 exposure but also that of their household. Due to the effects of structural racism, such that more non-Hispanic Black individuals are essential workers, COVID-19 mortality was higher among non-Hispanic Black persons in comparison to non-Hispanic White persons (26, 28, 29).

### Health Care

Because of limited or no high-quality, affordable broadband internet access, low-income communities, rural communities, the elderly with low digital literacy, and groups who have been historically marginalized are unable to fully benefit from contemporary evidence-based digital health services and tools. The COVID-19 pandemic accelerated individual and health system adoption of digital health tools for healthcare services (30, 31), in alignment with drastic limitations of in-person healthcare services and transitioning to virtual health service delivery that relies entirely on internet access (32). However, even though a study by the Department of Health and Human Services (HHS) revealed that there was a 63-fold increase in the use of telehealth services by White beneficiaries, Black beneficiaries were 2% less likely than whites to use telehealth services (33). Other studies show that individuals living in rural communities, the elderly, Black and Hispanic individuals, and low-income earners have been less likely to use telehealth services, predominantly due to limited/poor access to broadband internet (34, 35). These communities have greater difficulty navigating the healthcare system to find a preferred healthcare professional who is available, affordable (e.g., in-network), and accessible using a compatible device for telehealth visits and with sufficient broadband internet access. For example, lack of high-speed internet access has significantly affected the ability of Black individuals as compared to White individuals to access healthcare professionals (36).

Communities more likely to have poor internet access have experienced a decline in healthcare visits overall during the pandemic (35), but also a decline in the quality of their virtual visits if used. Because of task burden on mobile data and resultant slow internet speeds, families must choose and prioritize certain tasks over others (37). This translates into a barrier to accessing healthcare services when families choose to forgo health visits to prioritize, for example, schooling (37). Inconsistent medical check-ups are associated with high morbidity and mortality (38) and these consequences are likely to be more pronounced in marginalized groups who have a higher chronic disease burden in the absence of, but especially also during a pandemic. A recent study found that missed appointments was associated with increased all-cause mortality among those with chronic health conditions, particularly among patients with chronic mental health conditions (38).

Poor-quality internet limits families' ability to access healthcare services because of poor-quality audio or video during interactions with their healthcare professionals. Multiple interruptions from slow internet speeds during video telehealth visits interferes with rapport building, hinders patient comprehension, and frequently results in switching to telephone calls which is associated with poorer comprehension compared to video visits (39, 40). Additionally, because families may need to ration their data or internet usage, privacy may not always be able to be maintained for virtual visits. Special populations, such as individuals with mental health disorders, may be made even more vulnerable to poor mental health because they may be less likely to afford internet access as a result of unemployment and possible homelessness. This, in combination with the COVID-19 driven global rise in mental health disorder is likely to contribute to worsening of mental health care service disparities.

## Strategies to Expand Broadband Internet Access

**Figure 1:**
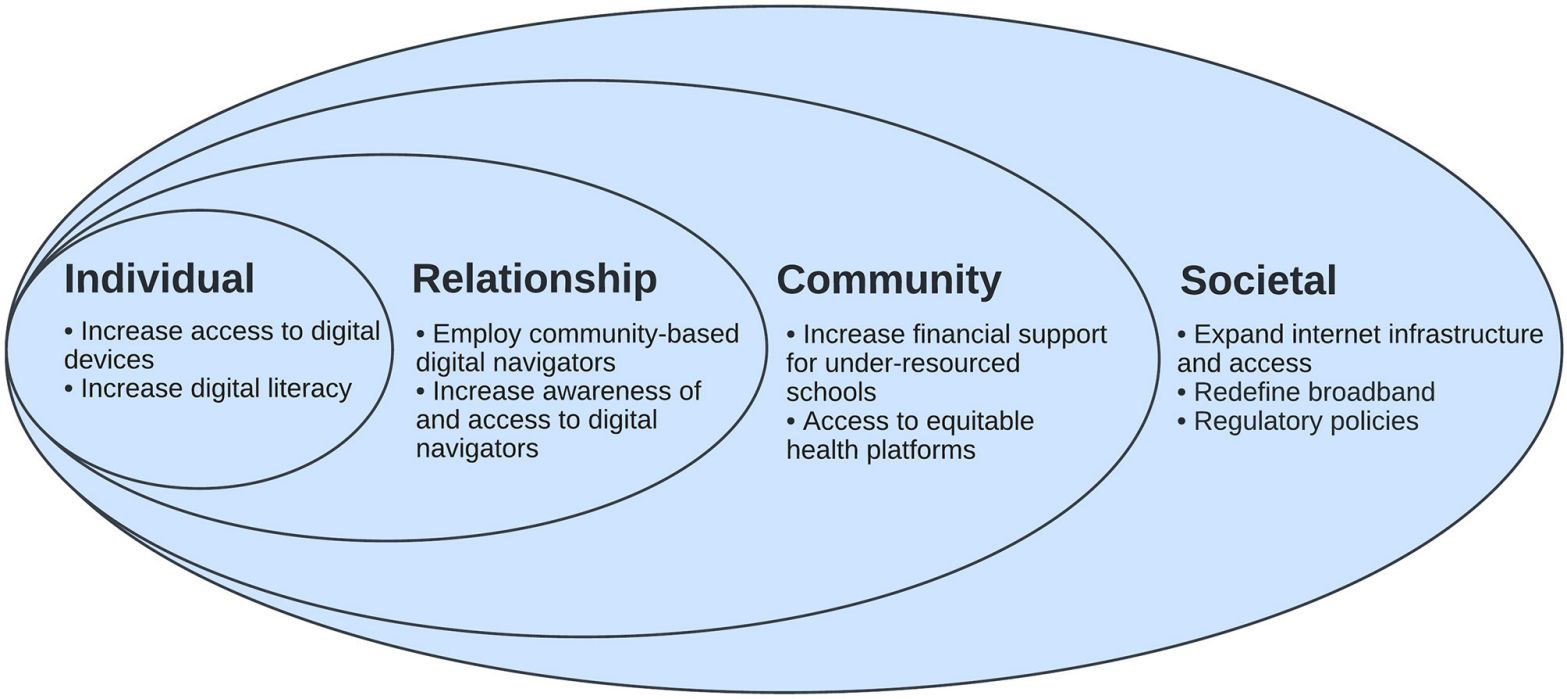
Socio-Ecological Model for Addressing Digital Redlining.

Broadband internet access is a SDH (4), intersecting and exacerbating other SDHs such as access to education and employment opportunities and access to high-quality healthcare services. The Social-Ecological Model can offer a clear roadmap of potential solutions to address digital redlining and expand broadband access to communities that have been structurally marginalized and minoritized. The Social-Ecological Model is a framework that acknowledges multiple levels of influence, guiding the development of solutions toward equitable access to broadband internet (41). We outline short- and long-term strategies with action points to address digital redlining (Table 1), including strategies at the societal, community, relationship, and individual levels (Figure 1).

**Table 1:** Key solutions and action points for addressing digital redlining.

**Socio-ecological domain**	**Key solutions**	**Action points**
Individual	1. Increase access to digital devices	• Establish programs (local, state, and federal) that subsidize cost of digital devices for individuals/populations that cannot afford these devices. • Health systems can develop digital health platforms that are accessible across multiple mobile operating systems and types of mobile devices.
	2. Increase digital literacy	• Providers and health systems should assess the level of digital literacy of their patients, i.e., ask if patients have clarity on how to use their devices for health-related purposes such as signing in for a doctor's visit, scheduling a lab test, reviewing their test results, sending a message to their provider. • Create handouts and videos that can walk patients through steps needed for digital device use.
Relationship	1. Employ community-based digital navigators	• Each clinic and hospital service should have access to a digital navigator who is available to explain to a patient, if needed, steps to using their mobile device or computer in accessing services. • The Information Technology (IT) department in each health system should be available at all times to respond to questions that patients may have about using their devices to access services. For systems or clinics without IT departments, a plan for accessing a digital navigator after hours should be in place. • Train current staff to provide services relating to digital navigation of technology for health services.
	2. Increase awareness of and access to digital navigators	• Part of the initial assessment and orientation to a clinic or hospital service should include information provided to the patient about how to access digital navigators. • Information about accessing digital navigators should be easily found on hospital/clinic websites.
Community	1. Increase financial support for under-resourced schools	• Local and state governments should include in their school budget funds for digital devices that includes internet access.
	2. Access to equitable health platforms	• Create digital health platforms that are accessible across multiple mobile and computer operating systems
Societal	1. Expand internet infrastructure and access	• Funding should be provided to internet companies to install adequate internet infrastructure in areas that are lacking, including cell towers, fiber, fixed wireless, digital subscriber lines (DSL), or cable • Expand WiFi hotspots to provide free WiFi for individuals who primarily access the internet using their mobile devices. • Subsidized cost of WiFi for specific individuals who cannot afford it.
	2. Redefine broadband	• Continuously review if the current definition of reliable high-speed internet meets current individual needs and update as needed. • Monitor internet maps to assess if residents are connected at the minimum requirement for high-speed internet.
	3. Regulatory policies	• States should use the Federal Communications Commission definition of reliable high-speed internet (download speeds of at least 25 Mbps and upload speeds of at least 3 Mbps). • Create regulations that ensure Internet Service Providers build infrastructure that meets the minimum requirement for high-speed internet.

### Societal Strategies

Societal strategies with high potential to increase equity in internet access are federal changes in the definition of broadband, broadband infrastructure expansion, and telecommunications regulatory policies. Redefining broadband access is a short-term solution that addresses inequities in the quality of broadband which limits access. The Federal Communications Commission (FCC), the agency responsible for defining broadband, defines it as 25 megabits per second download and three bits per second upload. This definition sets the standard for broadband access. However, this definition is misaligned with the current needs and how people use internet today. Broadband access is central to societal functions as aforementioned, such as supporting access to education, employment, health care, and socialization (42, 43). The current broadband standard set by the FCC is insufficient to provide quality access given how integral broadband is to our daily lives (44) and serves to mask racial and ethnic inequities in quality of broadband access which matters for broadband to be useable.

The expansion of broadband network infrastructures is a critical long-term strategy, particularly for rural and Tribal areas (45–47). Some urban areas still lack infrastructure; however, evidence shows that rural and tribal communities account for the majority share of households that lack broadband access (46, 48). The lack of infrastructure barring these communities from accessing specialty healthcare services, job opportunities, and opportunities for educational growth have implications for health and wellbeing (5, 49). Beyond broadband access, building infrastructure that allows for expansion of internet access (e.g., WiFi), particularly if free, will go a long way in ensuring equitable access particularly in areas where consumers rely predominantly on use of mobile phones for internet access. Polices that promote digital health equity will decrease the likelihood of disparities arising from digital redlining. The recently signed Infrastructure, Investment and Job Act (IIJA), is a step in the right direction (50).

A third societal-level solution is to reinstate policies regarding net neutrality, which would require internet service providers (ISPs) to provide access to website content at the same speed and under the same conditions (51). These policies would result in broadband service providers treating all services equally; thereby preventing companies from providing different levels of service driven by factors such as the quality of internet speeds or location. The ability of providers to distinguish levels of service, tied to different costs, creates a structure in which usable broadband is unaffordable to groups that have been structurally marginalized and minoritized (52). This structure perpetuates inequities in access broadly and most importantly, access to quality broadband. This also limits growth or emergence of new broadband providers which could disrupt the monopolization of broadband service and lead to lower cost (52).

There has been recent bipartisan effort to invest in affordable broadband in the United States (53). Current efforts such as President Biden's $65 billion investment earmarked for broadband infrastructure (54), the State of New York passing a law that requires all ISPs to offer high-speed internet plans to low-income families for $15 a month (55), Representative Yvette Clarke (NY) introducing an anti-digital redlining act (56), and the FCC's Digital Health Symposium on advancing broadband connectivity as a social determinant of health (57) are great attempts to address this issue.

### Community Strategies

At the community level, solutions that focus on schools and neighborhoods are key to achieving progress. The 1996 federal E-rate program, which made telecommunications and internet access more affordable to schools, provided a basic level of broadband connectivity (58). The common use of digital tools to facilitate learning and prepare students to function in an increasingly digital society makes access to quality broadband paramount. A 2014 benchmark set by the FCC recommended schools have an internet speed of 1 Mbps per student to support digital learning (59, 60). Only 33% of America's schools meet this goal (61) and likely to be lower given the rapid increase in digital teaching and learning due to the COVID-19 pandemic has outpaced broadband growth and quality (61, 62). Building internet infrastructure in school locations and allocating funds specifically toward expanding internet access will increase the likelihood that schools can meet and exceed this goal.

Additionally, the increase in the number of devices demands more robust broadband infrastructure at schools. Less resourced school districts and those that serve children of color are more likely to face these challenges (43, 46, 62). Increased financial and technical support for school districts and re-examination of provisions of the E-rate program are needed to increase equity. School districts also experience hidden costs associated with providing broadband internet access, like network security and management. State support can help ensure that all districts have adequate and high-quality access. Solutions that expand broadband internet access in certain neighborhoods could also be impactful in reducing health inequities, for example, in rural neighborhoods. Existing evidence demonstrates the success of public-private partnership solutions in providing broadband access to rural communities (45, 63). The creation and execution of a municipal broadband access strategy that provides access to all members of a community is another potential solution. In addition, we must create digital health platforms that are accessible across multiple mobile and computer operating systems.

### Relationship Strategies

Solutions at the relationship level are important to facilitate the diffusion of information relevant to digital access. As many transactions and interactions have moved online *via* digital collection tools and apps (i.e., registering for classes, searching for jobs, and accessing materials for school, job performance, or health), people often struggle to engage with information and communication technologies. For example, digital navigators, are individuals who can work with community members to increase digital inclusion and equity through sustained, direct interaction with individuals (64). They can be individuals who work in the social services sector, based within community organizations, and have the skills to facilitate community residents' access to online services and information. Digital navigators provide guidance on access to social needs, such as food, transportation, education, and health care; facilitate skills in app use for telehealth services or other myriad online services (64, 65). However, a key limitation currently is the lack of widespread employment of community-based digital navigators. Roles for digital navigators can be embedded within local public health departments and libraries to make them widely accessible to communities.

### Individual Strategies

Increasing access to digital devices and digital literacy are individual-level solutions that are critical to narrowing the digital divide. Relevant solutions should leverage school infrastructures to establish 1:1 device programs. These are programs where schools or districts provide each student with a device (e.g., tablet or computer). As of 2019, 51% of schools did not have a 1:1 device program (66). Providing 1:1 devices will help to standardize access to reliable online learning and increase students' families' exposure to digital tools (67, 68). Investment in digital literacy is a second area for targeted solutions that address digital redlining. Digital literacy is the ability to use information and communication technologies to find, evaluate, create, and communicate information, requiring both cognitive and technical skills (69). A 2018 report showed that Black adults were twice as likely as White adults to have limited digital literacy (60). Findings also show disparities according to rural residence and show that digital illiteracy is more prevalent in rural areas (49). Progress in digital literacy can be achieved with investments in digital navigators, community programming in areas of high need, and by leveraging educational systems to reach surrounding communities that are under-resourced (64, 65, 70, 71).

## Conclusion

The practice of digital redlining has contributed to the COVID-19 pandemic disproportionately affecting Black and Brown communities, precipitated by the rapid transition to virtual platforms for education, occupational and health purposes. Minoritized communities that have experienced disinvestment in necessary resources, such as broadband internet, have seen higher COVID-19 infection and mortality rates than their White counterparts (6). Individual strategies alone are insufficient to overcome the systemic inequities that are enumerated throughout this perspective piece. A synergistic multi-level approach, engaging community members about the strengths and needs of their communities must be employed in all efforts to make access to broadband internet equitable and decrease the negative impact of digital redlining exacerbated by the COVID-19 pandemic.

## Data Availability Statement

The original contributions presented in the study are included in the article/supplementary material, further inquiries can be directed to the corresponding author.

## Author Contributions

TM, CO, TL, and KW conceptualized the topic for this perspective piece. KA drafted the initial version of the Socio-Ecological Model for Addressing Digital Redlining which was further refined by a graduate student assistant (see Section Acknowledgments). All authors conducted literature searches and contributed to the writing, editing, and approval of the final version.

## Funding

TM and KW were supported by funding from the National Library of Medicine (NLM) Under Award Number R01LM013477. KW was also supported by NLM award G08LM013801 and Genentech, Inc. G-89371. KA was supported by CTSA Grant Number KL2 TR001862, NIDA Supplement 3UH3DA050251-03S1, and Fund to Retain Clinical Scientists at Yale, sponsored by the Doris Duke Charitable Foundation award #2020145, and the Yale Center for Clinical Investigation. CO was supported by National Heart, Lung, and Blood Institute K01HL145347.

## Conflict of Interest

TM is a member of the Clinical Diversity Advisory Board at Woebot Health, and Digital Wellbeing Advisory Board at Peer Health Exchange. KW received funding from Genentech, Inc. TL reports consulting fees from Plushcare, Inc. and JMIR Publications, Inc. Woebot Health, Peer Health Exchange, Genentech, Plushcare, and JMIR Publications were not involved in the writing of this article or the decision to submit it for publication. The remaining authors declare that the research was conducted in the absence of any commercial or financial relationships that could be construed as a potential conflict of interest.

## Publisher's Note

All claims expressed in this article are solely those of the authors and do not necessarily represent those of their affiliated organizations, or those of the publisher, the editors and the reviewers. Any product that may be evaluated in this article, or claim that may be made by its manufacturer, is not guaranteed or endorsed by the publisher.
